# Comparison of low density and high density pedicle screw instrumentation in Lenke 1 adolescent idiopathic scoliosis

**DOI:** 10.1186/s12891-017-1695-x

**Published:** 2017-08-02

**Authors:** Mingkui Shen, Honghui Jiang, Ming Luo, Wengang Wang, Ning Li, Lulu Wang, Lei Xia

**Affiliations:** 1 Institute of Spinal Deformity, the First Affiliated Hospital of Zhengzhou University, Zhengzhou, People’s Republic of China; 2 Department of Orthopaedic Surgery, Central Hospital of Wuhan, Tongji Medical College, Huazhong University of Science & Technology, Wuhan, People’s Republic of China

**Keywords:** Adolescent idiopathic scoliosis, Implant density, Low density, High density

## Abstract

**Background:**

The correlation between implant density and deformity correction has not yet led to a precise conclusion in adolescent idiopathic scoliosis (AIS). The aim of this study was to evaluate the effects of low density (LD) and high density (HD) pedicle screw instrumentation in terms of the clinical, radiological and Scoliosis Research Society (SRS)-22 outcomes in Lenke 1 AIS.

**Methods:**

We retrospectively reviewed 62 consecutive Lenke 1 AIS patients who underwent posterior spinal arthrodesis using all-pedicle screw instrumentation with a minimum follow-up of 24 months. The implant density was defined as the number of screws per spinal level fused. Patients were then divided into two groups according to the average implant density for the entire study. The LD group (*n* = 28) had fewer than 1.61 screws per level, while the HD group (*n* = 34) had more than 1.61 screws per level. The radiographs were analysed preoperatively, postoperatively and at final follow-up. The perioperative and SRS-22 outcomes were also assessed. Independent sample *t* tests were used between the two groups.

**Results:**

Comparisons between the two groups showed no significant differences in the correction of the main thoracic curve and thoracic kyphosis, blood transfusion, hospital stay, and SRS-22 scores. Compared with the HD group, there was a decreased operating time (278.4 vs. 331.0 min, *p* = 0.004) and decreased blood loss (823.6 vs. 1010.9 ml, *p* = 0.048), pedicle screws needed (15.1 vs. 19.6, *p* < 0.001), and implant costs ($10,191.0 vs. $13,577.3, *p* = 0.003) in the LD group.

**Conclusions:**

Both low density and high density pedicle screw instrumentation achieved satisfactory deformity correction in Lenke 1 AIS patients. However, the operating time and blood loss were reduced, and the implant costs were decreased with the use of low screw density constructs.

## Background

Adolescent idiopathic scoliosis (AIS) is the most common type of spinal deformity, afflicting the physical and mental health of adolescents; its incidence is 1–3% among 10–16-year-olds [1, 2]. The morbidity of Lenke 1 AIS, which is regarded as the most prevalent type and is defined as a structural main thoracic curve with non-structural proximal thoracic and thoracolumbar/lumbar curves, is 40% [3].

Pedicle screw construct systems have been increasingly popular for treating patients with spinal deformities, [4–7] and a significant correlation between the implant density and major curve correction has been reported [8–10]. However, substantial research has shown that low density (LD) screw constructs can provide similar radiographic and clinical outcomes [11–15]. Therefore, whether LD or high density (HD) screw constructs are better for AIS patients remains a subject of debate.

Previous studies have demonstrated that thoracic pedicle screw constructs could further improve the correction of spinal deformities compared with traditional hook and hybrid constructs [10, 16–19]. However, the use of fewer pedicle screws indicated a reduction of hospital expenses and risk of neurologic complications. If neurological complications or spinal cord injuries occur, the consequences could be disastrous.

The purpose of this study is to compare LD and HD pedicle screw patterns by radiographic, perioperative and Scoliosis Research Society (SRS)-22 outcomes in Lenke 1 AIS patients. We hypothesize that there would be no significant differences in deformity correction between LD and HD instrumentation, the treatment cost of LD could be reduced, and there would be decreased risk as fewer pedicles are implanted in the LD instrumentation.

## Methods

This retrospective study was approved by the Institutional Review Board of the participating hospital system. The medical records and radiographic outcomes of AIS patients were retrieved from a single institution from February 2009 to June 2013. All data were collected under a unified standard, and a standardized radiographic measurement was performed by a trained spine surgeon.

The inclusion criteria were as follows: (1) Lenke 1 AIS diagnosis; (2) main thoracic (MT) curve of more than 40° and less than 80°; (3) posterior spinal fusion with all pedicle screw constructs; (4) absence of a thoracoplasty; and (5) at least 2 years’ follow-up in radiographic and SRS −22 outcomes. The exclusion criteria were as follows: (1) previous spine surgery; (2) hooks or wires were used; and (3) pedicle subtraction osteotomy, vertebral column resection, or vertebral column decancellation techniques. Ultimately, sixty-two patients (39 females and 23 males) were included in this study.

**Fig. 1:**
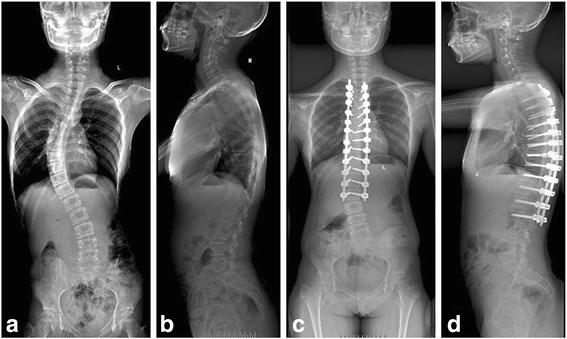
A high-density pedicle screw construct was used. Preoperative standing anteroposterior (**a**) and lateral radiographs (**b**). Final follow-up standing anteroposterior (**c**) and lateral radiographs (**d**)

**Fig. 2:**
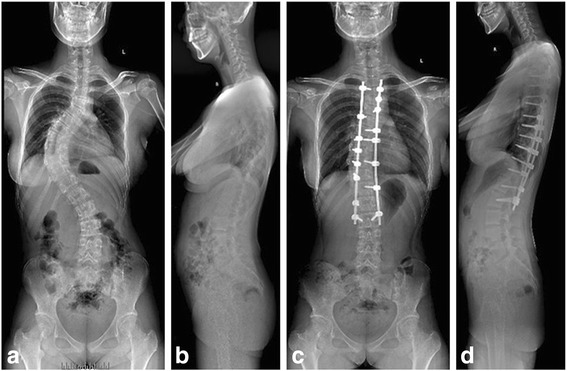
A low-density pedicle screw construct was used. Preoperative standing anteroposterior (**a**) and lateral radiographs (**b**). Final follow-up standing anteroposterior (**c**) and lateral radiographs (**d**)

During the intraoperative period, all pedicle screws were placed by a free-hand technique and confirmed with a C-arm before a senior surgeon placed a rod; all procedures were performed at a single institution. The implant density was defined as the number of fixation screws divided by the number of available anchor sites within the main curve [20]. Patients were divided into two groups according to the average screw density: the HD group was defined by an implant density above the mean number of screws per level for the entire cohort (>1.61 screws/level) (Fig. 1), while the LD group was defined by <1.61 screws/level (Fig. 2). The preoperative, postoperative and latest follow-up (average, 3.2 years; range, 2–5 years) radiographic outcomes were analysed with coronal and sagittal parameters. The perioperative outcomes and SRS-22 scores were also compared between the two groups.

### Surgical technique

After successful anaesthesia, patients were placed in the prone position. Using a midline incision, anatomical exposure of the spine was performed using a subperiosteal dissection of the paraspinal muscles. After confirmation of the location of the bilateral vertebral pedicles, screws were placed in an anatomic position. The two groups differed in the number of pedicle screws. The correction manoeuvres were the same for both groups. On the concave side, distraction was performed after single rod rotation. On the convex side, compression was performed after inserting the implant rod. The two-step locking caps were tightened. Allograft bone material and the disposed laminae and transverse processes were used for fusion.

### Radiographic, Perioperative and SRS-22 outcome measurements

Radiographic outcomes included assessments of the patients’ Risser grade [21], vertebral rotation index, convex-Bending Cobb angle, curve flexibility, lumbar spine modifier (A/B/C), thoracic sagittal profile, MT Cobb angle, thoracic kyphosis (T5-T12), lumbar lordosis (T1-L5), proximal junctional kyphosis, apical vertebral translation, and thoracic trunk shift in the preoperative, 2-week postoperative, and final follow-up periods. In addition, the change in the MT Cobb angle, correction rate of the MT curve, and loss of the MT Cobb angle were collected during the 2-week postoperative course and at final follow-up.

Perioperative records were reviewed to determine the operating time, blood loss, blood transfusion, hospital stay, implant costs, number of fused levels, number of screws, cross-link number, and screw density. We also assessed the SRS-22 scores preoperatively and at final follow-up.

### Statistical analysis

Data are presented as the mean ± standard deviation, and statistical analysis was performed using IBM SPSS Statistics v.21.0 (IBM Corp., Armonk, N.Y., USA). If collected data were distributed as the normality and equality of variances, independent sample *t* tests were used to compare the two groups’ baseline characteristics, radiographic and perioperative outcomes, and SRSS-22 scores. If not, the Kruskal-Wallis rank sum test and the Spearman rank correlation test were used. Statistical testing was two-sided, and a *p*-value <0.05 was considered statistically significant.

## Results

Sixty-two consecutive Lenke 1 AIS patients were ultimately included in this study (LD: *n* = 28; HD: *n* = 34). In the LD group, there were 17 females and 11 males, and the age at surgery was 14.2 ± 2.4 years. In the HD group, there were 22 females and 12 males, and the age at surgery was 14.8 ± 1.9 years. Based on a comparison of these two groups, there were no significant differences in age, Risser sign, MT Cobb angle, convex-Bending Cobb angle, flexible index, vertebral rotation index, apical vertebra translation, thoracic kyphosis, lumbar lordosis, and proximal junctional kyphosis. The baseline characteristics of the two groups are displayed in Table 1 **.**

**Table 1:** The baseline characteristics of the two groups

Variable	Low implant density	High implant density	(*p*-Value)
Modifiers (A/B/C)	18/4/6	26/3/5	—
TSP (−1/N/+1)	2/20/6	3/26/5	—
Gender (F/M)	17/11	22/12	—
Age (y)	14.2 ± 2.4	14.8 ± 1.9	0.31
Riser sign	2.5 ± 1.3	2.7 ± 1.1	0.52
MT Cobb (°)	56.5 ± 11.4	52.7 ± 10.1	0.07
convex-Bending Cobb (°)	33.5 ± 10.8	29.4 ± 6.5	0.07
Flexibility (%)	41.2 ± 12.9	45.8 ± 12.2	0.16
VR (Nash-Moe)	1.9 ± 0.7	1.7 ± 0.7	0.28
AVT (mm)	38.2 ± 14.4	34.1 ± 18.2	0.338
TTS (mm)	21.1 ± 15.6	15.7 ± 9.3	0.096
TK (T5–T12)	24.9 ± 11.3	30.9 ± 13.9	0.07
LL (T12–S1)	−52.5 ± 8.1	−55.1 ± 11.3	0.33
PJK(°)	5.4 ± 3.6	6.1 ± 3.0	0.43

*TSP* thoracic sagittal profile, *−1* hypokyphosis, *N* normal; *+1* hyperkyphosis, *F* female, *M* male, *MT* main thoracic, *VR* vertebral rotation, *AVT* apical vertebra translation, *TTS* thoracic trunk shift, *TK* thoracic kyphosis, *LL* lumbar lordosis, *PJK* proximal junctional kyphosis

Table 2 shows the perioperative measures of the two groups. Compared with the HD group, decreased operation time (278.4 vs. 331.0 min, *p* = 0.004), blood loss (823.6 vs. 1010.9 ml, *p* = 0.048), pedicle screws (15.1 vs. 19.6, *p*<0.001), and implant costs ($10,191.0 vs. $13,577.3, *p* = 0.003) were found in the LD group. However, no significant differences were detected in the hospital stays (18.7 vs. 19.9, *p* = 0.16) and cross-link numbers (0.6 vs. 0.3, *p* = 0.06).

**Table 2:** The perioperative outcomes of the two groups

Variable	Low implant density	High implant density	(*p*-Value)
Operating time (minutes)	278.4 ± 37.4	331.0 ± 86.7	0.004
Blood loss (ml)	823.6 ± 212.1	1010.9 ± 450.9	0.048
Blood transfusion (ml)	538.2 ± 295.3	597.6 ± 421.8	0.532
Hospital stays (days)	18.7 ± 3.3	19.9 ± 3.5	0.161
Implant costs ($)	10,191.0 ± 3326.48	13,577.3 ± 4939.2	0.003
Fused levels	12.0 ± 1.6	10.3 ± 2.3	0.001
No. screws	15.1 ± 2.9	19.6 ± 4.3	<0.001
Screw density	1.3 ± 0.2	1.9 ± 0.1	<0.001
Cross-link	0.6 ± 0.8	0.3 ± 0.6	0.06

Table 3 presents the two-week postoperative and final follow-up radiographic outcomes in terms of the coronal and sagittal correction between the two groups. Compared with the HD group, an increased MT Cobb angle was found in the LD group at the two-week postoperative assessment (18.4 vs. 14.3 degrees, *p* = 0.046). No significant differences were found in the MT Cobb angle, change of the MT Cobb angle, apical vertebra translation, thoracic trunk shift, thoracic kyphosis, lumbar lordosis, and proximal junctional kyphosis. There was a similar correction rate in the MT curve based on comparison of the two groups at two weeks postoperatively (67.9% vs. 74.3%, *p* = 0.053) and final follow-up (65.0% vs. 69.1%, *p* = 0.275).

**Table 3:** The radiographic outcomes of the two groups

Variable	Low implant density	High implant density	(*p*-Value)
TWO WEEKS POSTOPERATION			
MT Cobb (°)	18.4 ± 8.0	14.3 ± 7.7	0.046
Change of MT Cobb (°)	37.9 ± 7.9	38.4 ± 7.0	0.759
Correction rate (%)	67.9 ± 12.3	74.3 ± 12.9	0.053
AVT (mm)	17.0 ± 10.1	15.2 ± 9.1	0.474
TTS (mm)	10.4 ± 7.5	11.1 ± 7.9	0.701
TK (T5–T12)	19.5 ± 10.7	24.3 ± 10.0	0.071
LL (T12–S1) PJK(°)	−49.3 ± 10.3 9.2 ± 5.2	−47.7 ± 10.5 9.2 ± 5.3	0.546 0.99
FINAL FOLLOW-UP			
MT Cobb (°)	18.3 ± 7.9	17.0 ± 8.4	0.550
Change of MT Cobb (°)	34.5 ± 9.9	37.7 ± 8.5	0.175
Correction rate (%)	65.0 ± 15.1	69.1 ± 14.5	0.275
Loss of MT Cobb (°)	1.9 ± 5.1	1.4 ± 4.7	0.702
AVT (mm)	14.4 ± 9.2	13.7 ± 9.1	0.781
TTS (mm)	11.1 ± 9.8	10.6 ± 5.9	0.786
TK Cobb (T5–T12, °)	22.3 ± 12.4	23.5 ± 9.8	0.658
LL Cobb (T12–S1, °) PJK(°)	−53.8 ± 8.3 10.2 ± 6.7	−53.4 ± 10.1 13.4 ± 7.7	0.865 0.086

*MT* main thoracic, *AVT* apical vertebra translation, *TTS* thoracic trunk shift, *TK* thoracic kyphosis, *LL* lumbar lordosis, *PJK* proximal junctional kyphosis

**Fig. 3:**
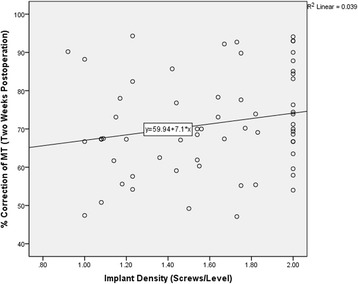
Correlation analysis between the implant density and correction rate of the MT curve for 2 weeks following the operation

**Fig. 4:**
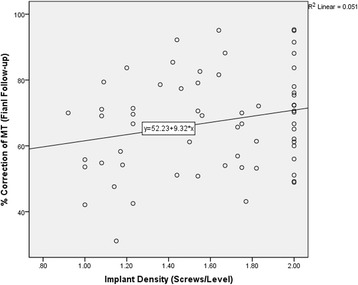
Correlation analysis between the implant density and correction rate of the MT curve for the final follow-up

The Spearman’s correlation coefficient was calculated to assess the relationship between the implant density and correction rate of the MT curve. The bivariate analysis showed no significant correlation between the implant density and correction rate of the MT curve at two weeks postoperatively (R^2^ = 0.039, *p* = 0.087) **(**Fig. 3 **)** and at final follow-up (R^2^ = 0.051, *p* = 0.136) **(**Fig. 4 **)**.

Table 4 describes the assessment of the quality of life of the two groups using the SRS-22 questionnaire preoperatively and at final follow-up. There were no significant differences between the two groups in terms of function/activity, pain, self-image, mental health and satisfaction.

**Table 4:** The SRS-22 questionnaire of the two groups

Variable	Low implant density	High implant density	(*p*-Value)
PRE-OPERATION			
Function/activity	3.7 ± 0.4	3.7 ± 0.3	0.797
Pain	4.4 ± 0.4	4.3 ± 0.5	0.419
Self-image	3.3 ± 0.4	3.2 ± 0.5	0.439
Mental health	3.9 ± 0.4	3.8 ± 0.4	0.224
Satisfaction	**-**	**-**	**-**
FINAL FOLLOW-UP			
Function/activity	3.8 ± 0.2	3.9 ± 0.2	0.293
Pain	4.3 ± 0.3	4.2 ± 0.3	0.647
Self-image	3.5 ± 0.3	3.6 ± 0.3	0.242
Mental health	4.0 ± 0.6	4.0 ± 0.6	0.166
Satisfaction	4.0 ± 0.6	4.0 ± 0.6	0.772

## Discussion

Pedicle screw instrumentation has become a common treatment for AIS patients [22]. Previous studies have demonstrated that pedicle screws could achieve safe and effective correction of the deformity compared with hybrid or hook constructs.

In this study, we compared the radiographic, perioperative and SRS-22 outcomes in Lenke 1 AIS patients using LD versus HD pedicle screw instrumentation. We found that there were no significant differences between the LD and HD groups in terms of the curve correction and SRS-22 outcomes. However, pedicle screw constructs using a low screw density not only achieved satisfied deformity correction in Lenke 1 AIS patients but also decreased the operative time, blood loss, and implant costs. Previous studies have investigated the relationship between the implant density and correction of AIS patients. Mac-Thiong et al. reported that adding fixation screws (an implant density of ≥70% in the main curve) was unlikely to result in significantly greater coronal correction of the main curve in posterior AIS surgery [20]. Li et al. found that a limited pedicle screw construct was equal to a consecutive screw construct in a randomized study, and there were no significant differences in the correction of the coronal and sagittal planes in Lenke 1 curves [23]. Kemppainen et al. reviewed 52 AIS patients with more than 2 years of follow-up and found that fewer screws not only achieved excellent curve correction, stability, and balance but also reduced the operative time and decreased the cost and risk [24]. Hosseini et al. published a study that used a series of 21 female patients who were treated with a novel technique and a lower implant density construct, achieving and maintaining a similar AIS correction as with current posterior fusion techniques [25]. In addition, Wang et al. used a three screw density pattern (low, preferred, and high screw density) in scoliosis patients and reported that there were no statistically significant results in terms of the curve correction or bone-screw force levels via biomechanical analysis [26]. In this study, our outcomes were supported by previous studies, and we found that LD and HD instrumentation were equally effective for major curve correction (*p* = 0.275) at the final follow-up.

In our study, decreased MT Cobb angle was found in the HD group at the two-week postoperative assessment (*p* = 0.046), which indicated that more pedicle screws achieved better deformity correction in the short-term postoperative period. It was possible that more pedicle screws could provide a stronger pull-out force during the single rod rotation procedure. However, a similar major Cobb angle was achieved with the LD and HD constructs by the final follow-up (*p* = 0.55). This could be caused by spontaneous correction of the major curve to allow for a well-balanced postoperative spinal column. In addition, sagittal plane alignment was obtained and maintained within normal parameters in the two groups, and no significant differences were found in thoracic kyphosis (*p* = 0.658), lumbar lordosis (*p* = 0.865), or proximal junctional kyphosis (*p* = 0.086) by the final follow-up. Liu et al. evaluated 77 Lenke type 1 AIS patients who underwent single-stage posterior correction and instrumented spinal fusion with pedicle screw fixation; they found that a high screw density on the concave side could provide better outcomes with respect to sagittal TK restoration [27]. Sudo et al. analysed 64 Lenke 1 AIS patients who were treated with posterior correction and fusion surgery, demonstrating that changes in thoracic kyphosis were significantly correlated with the screw density at the concave side (*r* = 0.351, *p* = 0.036), which was not the case on the convex side (*r* = 0.144, *p* = 0.40) [28]. Our findings contrasted with reports that increased sagittal correction was correlated with an increased screw density; we found that there were no significant differences in the coronal and sagittal Cobb correction.

As with the precognitive advantages found in LD instrumentation compared with HD instrumentation, a lower number of screws could significantly decrease the operating time (278.4 vs. 331.0 min, *p* = 0.004), blood loss (832.6 vs. 1010.9 ml, *p* = 0.048), and implant costs ($10,191.0 vs. $13,577.3, *p* = 0.003). Our study demonstrated that the LD instrumentation decreased the surgery time and cost without sacrificing the correction rate of the spinal deformity in the treatment of Lenke 1 AIS. Most importantly, a low implant density could somewhat diminish complications. Behrbalk et al. observed 21 Scheuermann kyphosis patients and concluded that the low screw density technique reduced the implant-related cost by 32%; meanwhile, it achieved safe and effective outcomes on a par with the high screw density technique [29]. In addition, Larson et al. explored the national inpatient database in the United States and found that by changing the HD screw pattern to an LD pattern, the total cost of AIS surgery would effectively be reduced by $11 million to $20 million [30]. In our study, no significant differences were found in the SRS-22 scores between the two groups at the time of the final follow-up. This conclusion could be helpful for both spinal surgeons and Lenke 1 AIS patients.

Neural complications in the surgical treatment of AIS could not be ignored. One patient in the HD group developed neurologic symptoms in the postoperative period, with a CT scan displaying problems with the T9 vertebral pedicle screw placement; thus, we removed the left internal fixation of T9. Diab et al. reviewed 1301 consecutive surgical cases of AIS and reported that the overall neurological complication rate was 0.69% [31]. A systematic review analysed 13,536 pedicle screws placed in 1353 paediatric patients, and the overall placement accuracy rate was 94.9% [32]. When adding more screws to the construct, the occurrence of neurological complications increased.

Some limitations must be addressed. First, because of the retrospective nature of this study, patients were not randomized to different implant densities according to the surgical procedure. The range of implant densities in the study can be attributed to the evolution of the surgical technique during the study. Second, the relatively small sample size was underpowered for identifying significant differences; a longer follow-up study will be performed to assess the maintenance of deformity correction.

## Conclusions

This study compared low density with high density pedicle screw instrumentation in terms of the clinical, radiological and SRS-22 outcomes in Lenke 1 AIS. The two groups achieved satisfactory deformity correction. However, the operating time and blood loss were reduced and implant costs were decreased with the use of low screw density constructs.

## Abbreviations

AIS: Adolescent idiopathic scoliosis
HD: High density
LD: Low density
MT: Major thoracic
SRS: Scoliosis Research Society
